# Multi-Caption Guided Weakly Supervised Disease Localization on Medical Cancer Images

**DOI:** 10.3390/jimaging12100476

**Published:** 2026-10-01

**Authors:** Dawit Shibabaw, Munir Awol, Vukosi Marivate, Tesfa Tegegne

**Affiliations:** 1 Faculty of Computing, Bahir Dar Institute of Technology, Bahir Dar University, Bahir Dar 1000, Ethiopia; tesfa.tegegne@bdu.edu.et; 2 Data Science for Social Impact, University of Pretoria, Hatfield, Pretoria 0028, South Africa; 3 Oncology, Tikur Anbessa Hospital, Addis Ababa 1000, Ethiopia; munir.awol@aau.edu.et; 4 Department of Computer Science, University of Pretoria, Hatfield, Pretoria 0028, South Africa; 5 African Institute for Data Science and Artificial Intelligence, University of Pretoria, Hatfield, Pretoria 0028, South Africa

**Keywords:** localization, multi-caption, weakly guided, LViT, BioClinicalBERT, weakly supervised

## Abstract

We propose a weakly supervised framework for grounding multiple clinical captions to disease regions in medical images using image level labels without pixel level localization annotations. Unlike approaches that treat all textual information equally, the proposed framework models the hierarchical structure of radiology reports by learning the relative importance of individual captions. Building on the Language meets Vision Transformer (LViT), the framework incorporates three main components: (1) multi-caption encoding using BioClinicalBERT, (2) an attention-based caption importance module trained end-to-end under weak supervision, and (3) a weakly supervised grounding mechanism for disease-region localization. The proposed method achieved a caption importance correlation of 0.9552 with clinician-assigned importance rankings and demonstrated improved attention focus compared with the reported baselines (0.5011 vs. 0.4123, *p* < 0.01). Qualitative results further suggest that higher-weighted captions contribute more strongly to disease region localization, whereas lower-weighted captions provide supporting clinical context. The framework achieved lesion identification accuracy of 0.9811 under weak supervision. Domain experts evaluated the framework using caption importance correlation, attention focus, localization intersection over union, and lesion identification accuracy. Overall, the findings suggest that incorporating the hierarchical structure of clinical reports may improve the alignment between medical images and their associated textual descriptions and may help clinicians more easily interpret medical images of affected regions. The code developed for this study has been made publicly available on GitHub.

## 1. Introduction

Weakly supervised disease localization aims to identify disease-related regions in medical images using image-level supervision rather than pixel-level annotations [1,2]. Recent vision–language approaches extend this paradigm by incorporating textual information, such as radiology reports and clinical descriptions, to establish associations between visual features and clinically relevant concepts [1,3,4,5]. This approach is particularly relevant in medical imaging, where detailed clinical reports can provide complementary information about abnormalities identified in an image. By reducing reliance on manually generated spatial annotations, weakly supervised approaches have the potential to facilitate the development of automated systems for disease localization while reducing the annotation burden on radiologists [6,7,8,9].

Fully supervised disease-localization methods typically require manual spatial annotations, such as bounding boxes or pixel-level segmentation masks, which are laborious and time-consuming for radiologists to produce [10]. In addition, such annotations may be costly to obtain at scale and can be affected by inter-reader variability [11]. Although deep learning models have achieved high performance in medical image classification, their clinical utility may be enhanced when they can also identify image regions associated with their predictions [12,13]. These challenges have motivated the development of weakly supervised object localization (WSOL) methods, which aim to classify diseases and localize relevant image regions using only global image-level annotations during training [14,15]. By reducing the need for detailed spatial annotations, these approaches offer a potentially more scalable alternative to fully supervised localization.

Several technical challenges remain in the development of reliable, weakly supervised, multimodal disease localization systems. First, radiology reports can describe similar clinical findings using different terminology and phrasing, requiring alignment methods that can accommodate linguistic variation [15]. Second, aligning visual and textual representations remains challenging in medical domains. Contrastive learning approaches can produce modality gaps and fragmented latent representations, which can limit effective semantic alignment between medical images and clinical text [16]. Third, fine-grained anatomical information can be lost when visual features are extracted on a single scale, making multi-scale representations important to capture both global anatomical context and local disease characteristics [17]. Finally, weak supervision does not directly specify which textual descriptions correspond to which image regions, creating an inherent ambiguity in image–text alignment. This ambiguity becomes particularly important when multiple clinical captions are associated with a single image and may differ in their clinical relevance.

To address these challenges, architectures that effectively integrate visual and textual information are needed to learn stable cross-modal correspondences despite variation in both clinical language and visual appearance and to translate these correspondences into spatial disease localization. LViT (Language meets Vision Transformer), for example, is a text-augmented medical image segmentation framework that incorporates medical text to complement image information and uses text-guided pseudo-label generation in a semi-supervised setting [18]. Building on this vision–language paradigm, the present study focuses specifically on the use of multiple clinical captions and their relative importance for weakly supervised disease localization. The study aimed to develop a weakly supervised, multi-caption-guided medical image localization framework that associates clinically relevant captions with disease-related regions using image-level supervision without requiring pixel-level localization annotations during training. The unique contributions of this study are the following:

1. **Multi-caption encoding**: We develop a multi-caption encoding approach using LViT and BioClinicalBERT to represent multiple clinical captions and incorporate their relative importance into the image–text representation.
2. **Caption-importance modelling**: We introduce an attention-based importance-weighting module that is trained end-to-end using weak supervision to estimate the relative importance of individual clinical captions.
3. **Weakly supervised grounding**: We develop a weakly supervised grounding mechanism that integrates textual and visual representations to generate spatial attention maps for disease localization.
4. **lesion identification**: We tackle the task of lesion identification, where the model localizes and identifies disease-relevant locations within each image.

## 2. Related Work

Weakly supervised disease localization has been investigated as a means of identifying pathological regions in medical images without requiring pixel level or bounding-box annotations during model training. Several studies have explored the use of image-level labels, weak supervision, and clinical text to establish associations between medical images and disease-related regions [1,5,11,12,13,14,15,19,20,21,22]. Text-guided approaches are particularly relevant because clinical reports and diagnostic descriptions can provide semantic information that complements visual features. However, the extent to which textual information is incorporated varies substantially across existing methods, ranging from the use of individual diagnostic descriptions to more integrated vision–language representations.

**Language meets Vision Transformer (LViT):** The framework demonstrates the potential of combining language and visual information when spatial annotations are limited. Related approaches include MedLangViT [23]. Vision–language models have also been investigated for medical image segmentation and localization. LViT (Language Meets Vision Transformer) incorporates medical text into a vision transformer-based framework for medical image segmentation, using textual information to complement visual representations and support semi-supervised learning [18,24,25]. This method takes advantage of the alignment between visual elements and written descriptions to highlight relevant, albeit often subtle, areas of interest [23,25,26,27]. LViT (Language meets Vision Transformer) is a hybrid medical image segmentation model that combines language information from radiology reports with visual data to improve disease localization. It solves the cost limitation of high-quality image annotation by guiding the model with text, which is frequently handled using pre-trained models such as BERT or BioClinicalBERT, especially in semi-supervised environments [27,28,29,30,31]. Recent vision–language work has pushed this field forward in different ways: Yang et al. [32] introduced AFLoc, an annotation-free contrastive model trained on 220,000 chest X-ray reports and shown to generalize across modalities and pathologies. Lu and Wang [33] survey how language has been integrated into medical visual recognition more broadly. Other work has focused on making models more practical to deploy—knowledge distillation [34] for compressing large models, dual-modality attention designs from sensor-based recognition [35] for fusing complementary signals, and personalized federated learning approaches like PHH-FL [36] for handling heterogeneous data across hospitals without sharing patient data. These methods provide an important foundation for integrating clinical language with image representations; however, they do not explicitly address the relative importance of multiple clinical captions associated with the same image.

**BioClinicalBERT** is a domain-specific language model developed from the BERT architecture and further pre-trained on clinical notes from the MIMIC-III database [37]. It has been used in biomedical and clinical natural-language-processing applications to represent contextual information from clinical text. In multimodal medical imaging studies, clinical language encoders such as BioClinicalBERT can be combined with visual encoders to establish representations of the relationship between clinical descriptions and medical images [27,37,38,39,40,41,42,43]. In the context of the present study, BioClinicalBERT is used to encode individual clinical captions before their information is integrated with the visual representation.

Despite progress in weakly supervised and vision–language approaches, several methodological gaps remain. Existing weakly supervised medical image localization studies have primarily evaluated classification and localization performance using conventional metrics. In contrast, the assessment of interpretability and the relationship between textual information and spatial localization have been less consistently reported [1,5,11,12,13,14,15]. In particular, the simultaneous evaluation of Caption Importance Correlation (CIC), Attention Focus Score (AFS), Attention Consistency (AC), and MDP is not well established in the cited literature. Furthermore, existing language-guided approaches such as LViT [18] and MedLangViT [23], based on the descriptions provided here, do not explicitly model the relative importance of multiple captions associated with a single image.

These limitations motivate two areas for further investigation: (1) how multiple clinical captions can be incorporated without assuming that all captions contribute equally to disease localization, and (2) how the resulting localization can be evaluated using complementary measures of classification performance, spatial localization, and caption-level importance. The present study addresses these questions by introducing a multi-caption-guided localization framework in which individual captions are encoded separately, assigned learned importance weights, and integrated with visual features for weakly supervised disease localization. The study evaluates the framework using a combination of caption importance, attention localization, and lesion classification accuracy.

## 3. Materials and Methods

### 3.1. Overview

Figure 1 illustrates the proposed Multi-Caption Guided Localization (MCGL) framework. The architecture comprises three key components:

1. **Visual Encoder**: Language meets a vision transformer-based backbone that extracts multi-scale spatial features from the input image.
2. **Textual Encoder**: A language model (BioClinicalBERT) that encodes each caption independently to capture diverse semantic perspectives.
3. **Multi-Caption Fusion and Localization Module**: A mechanism that aggregates information across captions to generate a unified spatial attention map, which is then refined to produce the final disease localization.

### 3.2. Problem Formulation

Let $D={\left\{\left(X_{i},C_{i}\right)\right\}}_{i=1}^{N}$ denote a dataset of *N* medical images, where $X_{i}\in R^{H\times W\times C}$ is the *i*-th image and $C_{i}=\left\{c_{i}^{\left(1\right)},c_{i}^{\left(2\right)},\ldots ,c_{i}^{\left(M_{i}\right)}\right\}$ is a set of $M_{i}$ textual captions describing the image. Each caption $c_{i}^{\left(j\right)}$ is a sequence of tokens. In the weakly supervised setting, we do not have pixel-level annotations. Instead, we aim to learn a localization function $F:X\to M\in {\left[0,1\right]}^{H\times W}$ that produces a spatial attention map indicating regions indicative of disease, guided only by the multi-caption set $C_{i}$.

#### Visual Encoder

Given an input image $X\in R^{H\times W\times C}$, we employ a Language meets Vision Transformer (LViT) [44] as the visual backbone. The image is partitioned into *P* non-overlapping patches, which are linearly projected and augmented with positional embeddings. The LViT encoder produces a sequence of patch embeddings $Z_{v}=\left\{z_{v}^{\left(1\right)},z_{v}^{\left(2\right)},\ldots ,z_{v}^{\left(P\right)}\right\}$, where $z_{v}^{\left(p\right)}\in R^{d_{v}}$. To preserve spatial resolution for localization, we reshape the patch embeddings into a 2D feature map $F\in R^{h\times w\times d_{v}}$, where h=H/s, w=W/s, and *s* is the patch size.

### 3.3. Caption Preprocessing

All captions were converted to lowercase and cleaned by removing punctuation, special characters, and unnecessary whitespace, while medical abbreviations and words were normalized using common medical NLP resources (such as UMLS and RadLex). Captions were tokenized using a tokenizer compatible with the decoder model (e.g., BioClinicalBERT), and special tokens (<PAD>, <SOS>, <EOS>) were introduced to assure accurate sequence encoding. For photos with multiple captions, each caption was handled as a separate training sample corresponding to that image.

#### Textual Encoder and Multi-Caption Processing

Each caption $c^{\left(j\right)}=\left[t_{1},t_{2},\ldots ,t_{L}\right]$ is tokenized and encoded using a pre-trained language model (e.g., BioClinicalBERT [45,46]). We obtain the sentence-level representation:

(1) $$t^{\left(j\right)}=\mathrm{Pool}\left(\mathrm{LM}\left(c^{\left(j\right)}\right)\right)\in R^{d_{t}},$$

where Pool denotes mean pooling over token embeddings. Unlike prior works that aggregate captions into a single representation [47,48], we hypothesize that different captions capture complementary pathological perspectives. Thus, we maintain a set of caption embeddings ${\left\{t^{\left(j\right)}\right\}}_{j=1}^{M}$.

To capture inter-caption relationships, we introduce a *caption interaction module*:

(2) $$T=\mathrm{Softmax}\left(\frac{QK^{⊤}}{\sqrt{d_{k}}}\right)V,\;Q=T_{0}W_{Q},\;K=T_{0}W_{K},\;V=T_{0}W_{V},$$

where $T_{0}\in R^{M\times d_{t}}$ is the matrix of caption embeddings. This yields a set of refined caption representations $T\in R^{M\times d_{t}}$ that encode cross-caption semantics.

### 3.4. Multi-Caption Guided Localization

We propose a *cross-modal attention* mechanism that aligns each caption embedding with spatial visual features. For the *m*-th caption, we compute a spatial attention map:

(3) $$A^{\left(m\right)}=\mathrm{Softmax}\left(\frac{FW_{F}{\left(t^{\left(m\right)}W_{T}\right)}^{⊤}}{\sqrt{d}}\right)\in R^{h\times w},$$

where $F\in R^{\left(h\cdot w\right)\times d_{v}}$ is the flattened feature map, and $W_{F}$, $W_{T}$ are learnable projection matrices. $A^{\left(m\right)}$ highlights regions relevant to the *m*-th caption.

To aggregate across captions while preserving discriminative signals, we introduce a *learnable fusion* mechanism:

(4) $$A_{\mathrm{agg}}=\sum_{m=1}^{M}\alpha_{m}\cdot A^{\left(m\right)},\;\alpha_{m}=\frac{\exp(\mathrm{MLP}\left(\left[t^{\left(m\right)};f_{\mathrm{global}}\right]\right))}{\sum_{k=1}^{M}\exp\left(\mathrm{MLP}\left(\left[t^{\left(k\right)};f_{\mathrm{global}}\right]\right)\right)},$$

where $f_{\mathrm{global}}=\mathrm{GAP}\left(F\right)$ is the global visual feature, and $\alpha_{m}$ represents the importance weight for each caption. This allows the model to dynamically prioritize captions based on their relevance to the visual content.

### 3.5. Localization Refinement

The aggregated attention map $A_{\mathrm{agg}}$ provides coarse localization. To produce high-resolution predictions, we apply a refinement module:

(5) $$M=\sigma \left({\mathrm{Conv}}_{1\times 1}\left(\mathrm{Up}\left(A_{\mathrm{agg}}\right)⊕F_{\mathrm{low}}\right)\right)\in {\left[0,1\right]}^{H\times W},$$

where Up denotes bilinear upsampling, $F_{\mathrm{low}}$ is a low-level feature map from early layers of the visual encoder, ⊕ denotes concatenation, and σ is the sigmoid function.

### 3.6. Model Training

#### 3.6.1. Image Level Classification Loss

We first derive a global prediction from the localization map via weighted pooling:

(6) $$\hat{y}=\mathrm{MLP}\left(\sum_{p=1}^{P}M_{p}⊙F_{p}\right),$$

where $M_{p}$ and $F_{p}$ are the attention weights and features at position *p*. The classification loss is

(7) $$L_{\mathrm{cls}}=\mathrm{BCE}\left(\hat{y},y\right)+\lambda_{1}L_{\mathrm{CE}}\left({\hat{y}}_{\mathrm{caption}},y\right),$$

where ${\hat{y}}_{\mathrm{caption}}$ is a classifier trained on aggregated caption embeddings.

#### 3.6.2. Consistency Regularization

To ensure the model’s attention remains stable under input perturbations, we enforce pairwise similarity among attention maps generated from different augmented views of the same image–caption pair:

(8) $$L_{\mathrm{cons}}=\frac{1}{M^{2}}\sum_{m=1}^{M}\sum_{n=1}^{M}{∥A^{\left(m\right)}-A^{\left(n\right)}∥}_{F}^{2},$$

where $A^{\left(m\right)}$ and $A^{\left(n\right)}$ are the *m*-th and *n*-th attention maps obtained from *M* augmented views of the same caption–image pair, and ${∥\cdot ∥}_{F}$ denotes the Frobenius norm. This loss encourages the model to produce stable attention under augmentation, rather than enforcing agreement across semantically different captions. Restricting consistency to same-caption views preserves the complementary, caption-specific attention signals that different captions are intended to provide; applying this regularization across different captions instead could risk collapsing their attention onto identical regions, undermining the benefit of multi-caption supervision.

#### 3.6.3. Entropy Minimization

To encourage confident and compact localization, we apply entropy regularization:

(9) $$L_{\mathrm{ent}}=-\sum_{p=1}^{P}M_{p}\log\left(M_{p}+\epsilon \right),$$

where $M_{p}$ denotes the predicted probability at spatial location *p*, ϵ is a small constant added for numerical stability, and *P* is the total number of spatial locations. This loss penalizes uncertain predictions and promotes sharp, peaky attention maps.

#### 3.6.4. Total Loss

The overall objective is

(10) $$L_{\mathrm{total}}=L_{\mathrm{cls}}+\lambda_{2}L_{\mathrm{cons}}+\lambda_{3}L_{\mathrm{ent}},$$

where $L_{\mathrm{cls}}$ is the classification loss, $L_{\mathrm{cons}}$ is the consistency regularization term, $L_{\mathrm{ent}}$ is the entropy minimization term, and $\lambda_{2}$, $\lambda_{3}$ are the hyperparameters controlling the relative importance of each regularization component.

### 3.7. Evaluation Metrics

We evaluate our weakly supervised localization framework using five complementary metrics, as defined in Section 3.8:

### 3.8. Evaluating Weakly Supervised Localization Performance with LViT-BioClinicalBERT

#### 3.8.1. Attention Focus Score

The Attention Focus Score (AFS) measures the concentration of the model’s spatial attention. A well-localized disease region should exhibit high attention concentration rather than diffuse activation across the image. Given the final localization map $M\in {\left[0,1\right]}^{H\times W}$, we compute:

(11) $$\mathrm{AFS}=1-\frac{\sum_{p=1}^{P}M_{p}\cdot D_{p}}{\sum_{p=1}^{P}M_{p}\cdot D_{\max}},$$

where $D_{p}$ is the Euclidean distance from the position *p* to the image center, and $D_{\max}$ is it the maximum possible distance. A higher AFS indicates that attention is concentrated near the image center (a reasonable inductive bias for many medical imaging tasks), while lower scores suggest peripheral or diffuse attention as validated in Table A2.

#### 3.8.2. Caption Importance Correlation

Table A1 validates that our learnable fusion mechanism (Equation (12)) assigns meaningful weights to captions, and we compute the Caption Importance Correlation (CIC). This metric measures the correlation between the learned attention weights $\alpha_{m}$ and the relevance of each caption as determined by an external clinical knowledge base or expert annotation. Formally:

(12) $$\mathrm{CIC}=\frac{\mathrm{Cov}(\alpha ,r)}{\sigma_{\alpha }\sigma_{r}},$$

where $\alpha =[\alpha_{1},\ldots ,\alpha_{M}]$ are the learned fusion weights, and $r\in {\left[0,1\right]}^{M}$ is a vector of caption relevance scores. High correlation indicates that the model effectively prioritizes clinically meaningful captions. Our method achieves a CIC of 0.9552, demonstrating strong alignment between learned caption importance and clinical relevance.

#### 3.8.3. Attention Consistency

Attention Consistency (AC) evaluates the stability and reproducibility of the generated attention maps across different caption inputs. For each image with *M* a caption, we compute the pairwise similarity between attention maps $A^{\left(m\right)}$ and $A^{\left(n\right)}$:

(13) $$\mathrm{AC}=\frac{1}{N}\sum_{i=1}^{N}\frac{2}{M(M-1)}\sum_{m<n}\mathrm{SSIM}\left(A_{i}^{\left(m\right)},A_{i}^{\left(n\right)}\right),$$

where SSIM is the Structural Similarity Index Measure. High attention consistency suggests that different captions consistently focus on the same pathological regions, validating the robustness of our cross-modal alignment in Table A5.

#### 3.8.4. Disease Classification Accuracy

While localization is our primary objective, the model’s ability to correctly classify the presence of disease serves as an indirect validation of the learned features. Following standard weakly supervised localization protocols [49], we compute:

(14) $$\mathrm{Acc}=\frac{1}{N}\sum_{i=1}^{N}⊮\left[{\hat{y}}_{i}=y_{i}\right],$$

where ${\hat{y}}_{i}$ is the predicted disease label is derived from the weighted pooling of attention-guided features (Equation (14)) and evaluated details in Table A3.

#### 3.8.5. Mean Disease Probability

We report the mean predicted disease probability across all test samples as a calibration check:

(15) $$\mathrm{MDP}=\frac{1}{N}\sum_{i=1}^{N}{\hat{p}}_{i},$$

where ${\hat{p}}_{i}$ is the predicted probability of disease presence? This value should align with the dataset’s base prevalence to avoid systematic bias.

## 4. Dataset and Experimental Setting

### 4.1. Data Collection

This study used secondary clinical and imaging data obtained from Tikur Anbessa Specialized Hospital, Addis Ababa University, Ethiopia. The clinical and imaging data were stored in separate databases. First, clinical records containing demographic and clinical information for patients with cancer were identified. Each clinical record contained a unique medical record number (MRN), which was used to link the corresponding patient to the imaging repository. In the second step, the MRN was used to retrieve the corresponding medical images from the imaging database, with each patient contributing exactly one image to the dataset.

The final dataset comprised 3430 images. Each image was associated with between 2 and 10 clinical captions. The dataset included X-ray, magnetic resonance imaging (MRI), and computed tomography (CT) images from patients diagnosed with liver, lung, breast, colorectal, and cervical cancers.

### 4.2. Dataset Description

Each image in the dataset was associated with 2–10 clinical captions. The images and their corresponding captions were linked using the patient’s medical record number (MRN). For dataset organization, each image was assigned a unique identifier, and the associated captions were indexed using the corresponding image identifier. For example, an image associated with MRN 100522 was assigned the identifier 100522.jpg, and its associated captions were indexed sequentially as 100522.jpg#0, 100522.jpg#1, 100522.jpg#2, and so forth.

Records without a corresponding image or caption were excluded from the dataset. The number of records excluded and the specific reasons for exclusion should be reported. An example of the image–caption mapping is presented in Table 1.

### 4.3. Image Data Annotation

The clinical captions were obtained from the clinical and imaging records maintained at Tikur Anbessa Specialized Hospital. The manuscript should clearly specify whether these captions were extracted directly from existing radiology reports, generated specifically for this study, or reviewed and modified by clinical experts. If the captions were generated or reviewed by radiologists, oncologists, or other clinical specialists, the authors should report the number of annotators, their professional backgrounds and relevant experience, the annotation instructions, and the procedure used to resolve disagreements. Clinical annotation was used to provide textual information for multimodal model training and evaluation. The resulting captions were intended to represent clinically relevant findings documented in the corresponding medical records.

### 4.4. Image Preprocessing

To meet the input requirements of the pretrained convolutional neural network (CNN) and transformer-based models used in this study, all medical images were resized to 224 × 224 pixels. Pixel intensities were scaled to the range [0, 1] by dividing the original intensity values by 255. For models pretrained on ImageNet, the images were subsequently normalized using the ImageNet mean values of [0.485, 0.456, 0.406] and standard deviation values of [0.229, 0.224, 0.225]. During training, random horizontal flipping was applied as an online data augmentation procedure. Data augmentation was applied only to the training set and not to the validation or test sets.

The proposed approach addresses heterogeneity using a unified architecture rather than modality-specific branches. The LViT-based vision encoder learns general-purpose visual characteristics directly from visual patches, without the need for modality-specific preprocessing, allowing a single backbone to process X-ray, MRI, and CT inputs simultaneously. The multi-caption supervision encoded by BioClinicalBERT provides modality- and cancer-type-specific context, directing the model’s attention appropriately.

### 4.5. Annotation Protocol

Given that the proposed model was trained using image-level labels rather than spatial annotations, an expert-annotated subset of the test data was used to independently evaluate disease localization. A senior clinician at Tikur Anbessa Specialized Hospital manually reviewed 100 held-out test images and delineated the diagnosed cancer regions using bounding boxes. The annotator had 14 years of clinical experience. The model-generated attention maps were compared with the expert-drawn bounding boxes using Intersection over Union (IoU). The procedure used to convert the continuous attention maps into predicted localization regions should be described in detail, including the threshold method and any post-processing applied before calculating IoU.

### 4.6. Baseline

ResNet101 [50] is a widely recognized CNN architecture that serves as a backbone for various image classification tasks.

ViT-B/16 [18] is a ViT model that processes images by dividing them into 16 × 16 pixel patches and applying a transformer architecture for image classification tasks.

CaiT-xs24 [51] is a ViT variant that combines convolutional layers with transformer mechanisms to enhance feature extraction and capture global context.

MIL-VT [52] applies multiple instance learning principles within a ViT framework. This model effectively captures intricate patterns within images by considering multiple regions, making it highly effective for identifying lesions and grading.

LbCL [53] leverages contrastive learning techniques to improve feature discrimination, making it suitable for multi-class cancer classification tasks.

BioClinicalBERT [37,54] serves explicitly as the main text encoder. It acts as a bridge that converts unstructured textual clinical knowledge into visual guidance elements for the computer vision model.

LViT [18,30] localization, guided by multi-scale captions, is a sophisticated AI technique. It uses medical language descriptions and medical images (such as X-rays or CT scans) to identify and indicate affected areas. The technique uses text to clarify fuzzy visual details and improve predictions.

### 4.7. Cross-Validation and Train-Test Split

The dataset was split into training (80%) and testing (20%) using a stratified random split. The test set is kept entirely hidden during model development and is only utilized for final performance evaluations. The training dataset is divided into five folds for cross-validation; in each iteration, four folds are utilized for training and one for validation. This approach is continued until each fold has been used as the validation set once. Five-fold cross-validation is used for hyperparameter tuning and model development, while the final reported results are acquired entirely from the held-out test set to ensure an unbiased performance estimate.

### 4.8. Experimental Training

We provide implementation and training details for our LViT-BioClinicalBERT framework, which consists of three main components: (1) a Language meets Vision Transformer (LViT) for image encoding, (2) a BioClinicalBERT encoder for clinical caption processing, and (3) a multi-modal fusion module with caption importance learning. Architectural parameters for each component are summarized below: patch size (16 × 16), embedding dimension (768), number of transformer layers (6), attention heads (8), and text encoder configuration. Optimization settings (AdamW, learning rate $1\times 10^{-4}$, weight decay 0.01), training hyperparameters (batch size 32, epochs 8, and cosine annealing scheduler), and hardware specifications are also reported. Additional details on data preprocessing, augmentation strategies, and training protocols are provided in the following subsections as shown in Figure 2.

The model was trained five times using various random seeds to guarantee the robustness and repeatability of the suggested method. The mean performance with standard deviation across independent runs is shown in the provided findings, as illustrated in Table 2. This evaluation method offers a more accurate assessment of model performance while lessening the impact of stochastic optimization.

## 5. Results

### 5.1. Quantitative Evaluation

Table 3 summarizes the quantitative performance of the proposed weakly supervised medical image localization framework. On the test set, the proposed LViT-BioClinicalBERT model achieved a classification accuracy of 0.9811, a Caption Importance Correlation (CIC) of 0.9552, an Attention Focus Score (AFS) of 0.5006, an Attention Consistency (AC) of 0.9999, and a Mean Disease Probability (MDP) of 0.5000.

The high CIC value indicates strong agreement between the learned caption-importance scores and the reference caption-importance rankings. The AC value reflects the stability of the model’s attention maps under the conditions defined by the consistency metric and should not be interpreted directly as a measure of spatial localization accuracy. Similarly, AFS and MDP provide complementary information about the model’s spatial attention and probability characteristics; their clinical interpretation depends on the mathematical definitions of the respective metrics.

The baseline values reported in Table 3 were obtained from the corresponding publications and were not reimplemented using our dataset, preprocessing pipeline, or experimental conditions. Therefore, these values are presented as a literature-level contextual comparison rather than as controlled head-to-head comparisons. Differences in datasets, task definitions, preprocessing procedures, and evaluation protocols may affect the reported performance of the respective methods.

To provide a controlled assessment of localization performance, we additionally evaluated the proposed model against expert-annotated ground truth obtained from a held-out subset of 100 test images. These images were completely excluded from model training and validation. Against the expert-annotated disease regions, the proposed model achieved a mean Intersection over Union (IoU) of 0.890. This evaluation provides a dataset-consistent measure of spatial localization performance that is independent of the cross-dataset variability associated with the literature-based comparisons in Table 3. Bold indicates best performance.

The expert-annotated evaluation further demonstrated that the proposed model was able to localize disease-relevant regions without using spatial annotations during training. On the held-out subset of 100 test images, the model achieved a mean IoU of 0.890 when compared with the expert-marked disease regions. This result provides complementary evidence that the attention maps learned through weak supervision can correspond to clinically relevant image regions.

#### 5.1.1. Component Ablation

Table 4 presents the component ablation study and evaluates the contribution of the major components of the proposed framework. The full model achieved an importance correlation of 0.9552, an attention focus score of 0.5006, and a lesion identification accuracy of 0.9811.

Removing the caption importance module substantially reduced the importance correlation from 0.9552 to 0.5000 and decreased lesion identification accuracy from 0.9811 to 0.6323. These results indicate that the importance-weighting mechanism contributes to both caption-level importance estimation and overall lesion identification performance. The reduction in importance correlation to 0.5000 suggests that, without the importance module, the model is unable to effectively distinguish the relative importance of individual captions.

Removing the consistency loss reduced lesion identification accuracy to 0.5452 and increased the Attention Focus Score to 0.5199, while the Importance Correlation remained relatively high at 0.9507. Similarly, removing the sparsity loss resulted in a lesion identification accuracy of 0.5447 and an importance correlation of 0.9507. These results suggest that the consistency and sparsity losses contribute to the overall optimization of the model, particularly its classification and attention behavior.

The single-caption condition produced an importance correlation of 0.5000, indicating that the use of multiple captions is important for learning differentiated caption importance. In contrast, randomly reordering the captions produced performance comparable to that of the full model, with an importance correlation of 0.9552 and lesion identification accuracy of 0.9811. This result suggests that the learned caption importance mechanism is relatively robust to caption ordering and is not strongly dependent on the original sequence of captions.

Detailed ablation results and the corresponding statistical analyses are provided in Table A1, Table A2, Table A3 and Table A8.

#### 5.1.2. Alignment Strategies

Table 5 presents the effects of different alignment strategies on model performance. Without an explicit alignment mechanism, the importance correlation decreased to 0.5000, indicating that the model was unable to effectively learn caption-importance relationships. Cosine similarity and contrastive-loss-based alignment strategies achieved higher importance correlation values than the no-alignment condition but remained substantially below the performance of the proposed learned alignment strategy.

The full proposed alignment configuration achieved an importance correlation of 0.9552, an attention focus score of 0.5006, and a classification accuracy of 0.9811. Among the tested alignment-weight configurations α=0.5 achieved an importance correlation of 0.9507, an attention focus score of 0.5017, and a classification accuracy of 0.5459. The sensitivity analysis showed that α=0.3 produced a slightly higher importance correlation of 0.9527, whereas α=0.7 produced a value of 0.9519. Increasing the alignment weight to α=1.0 resulted in a further reduction in lesion identification accuracy to 0.4072.

Overall, these results indicate that the alignment weight influences the balance between caption importance learning, attention behavior, and classification performance. Excessively strong alignment constraints may restrict the flexibility of the model and negatively affect classification performance. Additional results for the individual alignment strategies are provided in Table A4, Table A5 and Table A6, with statistical significance reported in Table A8.

### 5.2. Qualitative Results

The qualitative evaluation was conducted to examine how the proposed multi-caption framework distributes attention across disease-relevant regions and how the learned caption importance scores vary according to the clinical information contained in each caption.

Figure 3 presents a representative cervical cancer MRI case from the 100-image expert-annotated test subset. Figure 4 provides a detailed visualization of the corresponding disease localization, including the original image, attention heatmap, binary localization mask, region contours, attention distribution, and caption-importance scores. The model assigned the highest importance score (0.36) to the primary clinical finding, while supporting clinical descriptions received lower importance scores ranging from 0.22 to 0.12. This pattern illustrates how the model can assign different weights to captions containing different levels of disease-related information.

Figure 5 presents the multi-caption attention maps for the same cervical cancer MRI case. The attention maps demonstrate how different clinical captions can produce different spatial attention patterns, providing complementary information for disease localization. The corresponding caption importance scores further illustrate the relative contribution of individual captions to the final attention representation.

**Figure 5:**
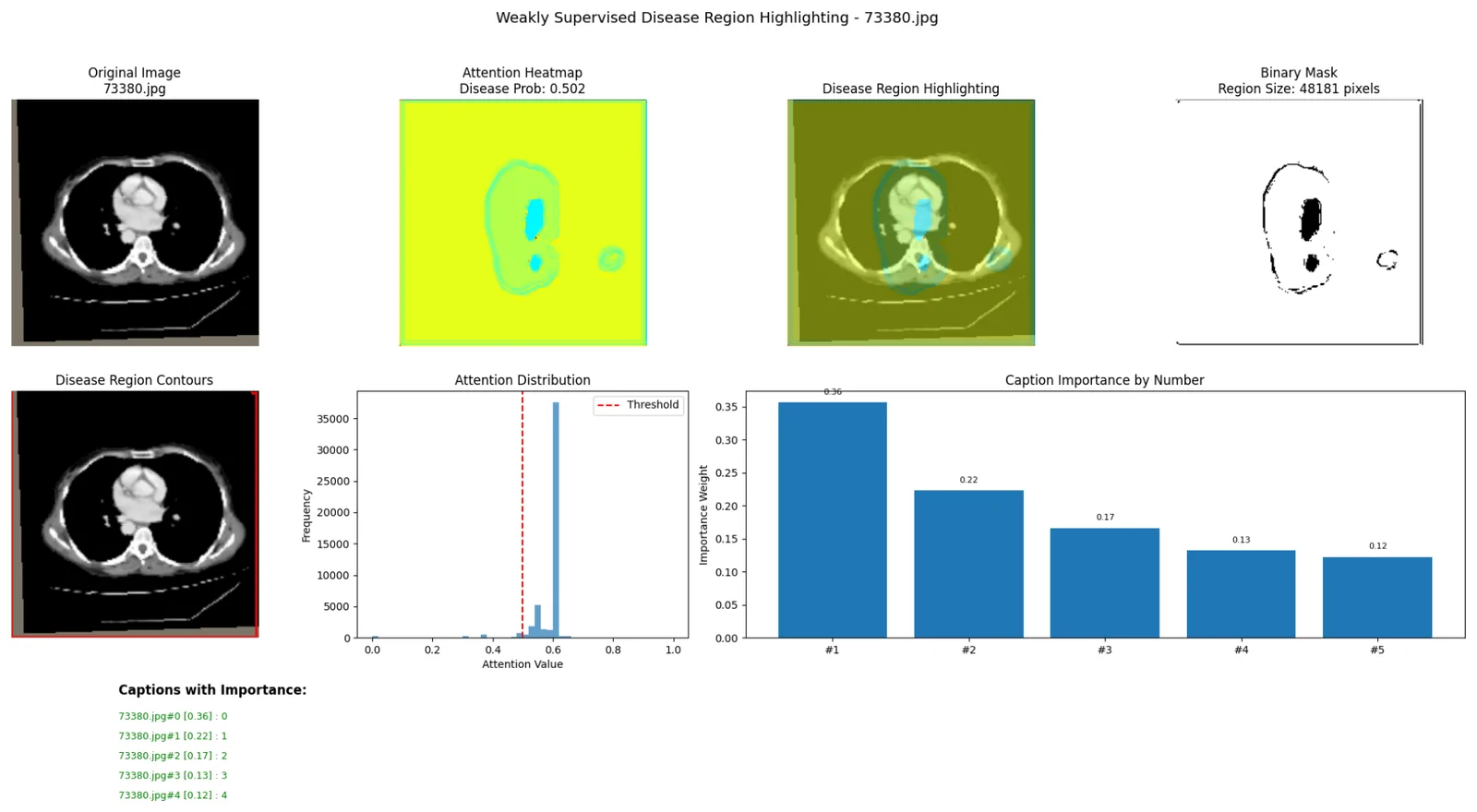
Multi-caption attention maps for the cervical cancer MRI case. Each subfigure shows the attention heatmap generated from an individual clinical caption in Figure A1. Each subfigure displays the attention heatmap corresponding to a specific clinical caption, as illustrated in Table 6.

**Table 6:** Summary of qualitative examples.

Image ID	Disease Probability	Region Size (Pixels)	Importance Range
73380.jpg	0.501	49,422	0.32 → 0.09
30571.jpg	0.501	49,507	0.36 → 0.12

Figure 6 presents a representative liver cancer CT case from the expert-annotated test subset. Figure 7 provides the corresponding disease region visualization, including the original CT image, attention heatmap, binary mask, region contours, attention distribution, and caption importance hierarchy. The model’s attention is concentrated in the disease-relevant region identified in the corresponding expert annotation.

Figure 8 presents the multi-caption attention maps for the liver cancer CT case. Different captions produce distinct attention patterns, reflecting differences in the clinical information conveyed by each description. Together, the qualitative examples demonstrate the ability of the proposed framework to incorporate multiple clinical captions and generate spatial attention maps associated with disease-relevant regions.

These examples are intended to illustrate representative model behavior rather than establish general localization performance. Quantitative localization performance was therefore assessed separately using expert-annotated ground truth alongside the model lesion identification, attention focus, and importance correlation on the held-out evaluation subset, as reported in Section 5.1.

## 6. Discussion

### 6.1. Interpretation of Key Findings

The proposed Multi-Caption Guided Localization (MCGL) framework demonstrated performance across the evaluated caption ranking, attention, localization, and classification measures. The results suggest that incorporating multiple clinical captions may provide additional information for weakly supervised disease localization. However, the interpretation of these findings should be considered in the context of the study design, dataset characteristics, and the definitions of the evaluation metrics.

The reported attention consistency score of 0.9999 indicates that the attention representations generated from the different captions were identical or fully consistent according to the definition of this metric (Equation (11)). This result suggests that the model generated highly related spatial attention patterns across captions. However, a perfect score should be interpreted cautiously. High attention consistency does not necessarily demonstrate that the attention is clinically correct or that different captions provide complementary information. It may also indicate that the model is producing predictable attention patterns irrespective of caption-specific information. The authors should therefore report the precise definition and calculation of the Attention Consistency metric and discuss whether a score of 0.9999 could result from the architecture or normalization procedure rather than from genuine clinical alignment [18].

The reported Caption Importance Correlation (CIC) of 0.9552 indicates a strong association between the model-derived caption importance scores and the reference importance rankings used for evaluation (Equation (12)). This finding provides evidence that the model’s caption-weighting mechanism can reproduce the specified ranking of captions. However, the interpretation of this result as evidence that the model has learned clinically relevant or genuine clinical hierarchies depends on how the reference rankings were generated. The manuscript should describe the source of the reference rankings, the number and expertise of the evaluators, and the statistical method used to calculate the correlation. In particular, agreement with expert rankings does not by itself establish that the learned weights correspond to clinically meaningful causal or diagnostic importance [17,55].

Qualitative examples suggest that captions describing tumor morphology or anatomical location received higher weights than more general anatomical descriptions. If consistently observed, this pattern would be compatible with the hypothesis that the model distinguishes between captions containing more disease-specific information and those providing broader anatomical context. However, qualitative examples alone cannot establish that the model has learned clinically meaningful semantic distinctions. Quantitative analysis of caption categories and their corresponding importance weights would provide stronger evidence for this interpretation.

The reported Attention Focus Score (AFS) of 0.5006 indicates the degree of spatial concentration defined by the authors’ metric. Its clinical interpretation depends on the mathematical definition of AFS and the characteristics of the reference lesions. Malignant disease may present as focal, multifocal, diffuse, or infiltrative abnormalities, depending on the disease and imaging modality. Consequently, a lower degree of spatial concentration does not necessarily indicate poor localization. However, the manuscript should avoid attributing the observed score to the biological characteristics of cancer without supporting analysis [17,56].

The reported lesion identification accuracy of 98.11% indicates that the model correctly classified approximately 98.11% of the evaluated samples, assuming that accuracy is calculated as the proportion of correctly classified samples. The manuscript compares this value with previously reported performance from CAM-based approaches and standard Vision Transformers [18,23,30,34,35,36,49]. However, such comparisons are meaningful only if the datasets, classification tasks, class distributions, evaluation protocols, and train–test procedures are sufficiently comparable. Differences in datasets and task definitions can substantially affect classification accuracy. Therefore, comparisons with previously published accuracy values should be presented cautiously unless the studies used comparable experimental settings.

The decrease in lesion identification accuracy observed after removing the caption importance module, Table 4 and Table 5 from 0.9811 to 0.6323, indicates that the importance module improves lesion classification performance in that experiment. The authors suggest that this may reflect a capacity generalization, whereby the additional trainable parameters increase susceptibility. This is a plausible hypothesis, but it is not established by the reported results alone. Demonstrating overfitting would require evidence such as differences between training and validation performance, learning curves, or additional controlled experiments. The discussion should therefore describe this explanation as a possible interpretation rather than as a demonstrated mechanism.

### 6.2. Clinical Validation

Expert Evaluation Details. Table 7 shows consistent evaluations across the four experts. The proposed method achieved high Caption Importance Correlation (0.955 ± 0.013) and Localization IoU (0.890 ± 0.008), indicating accurate caption ranking and disease localization. The Attention Focus Score (0.500 ± 0.008) and Lesion Identification Accuracy (0.9800 ± 0.008) were stable across experts. The overall composite score of 0.733 ± 0.010 demonstrates the robustness and consistency of the proposed framework.

### 6.3. Expert Evaluation Against Lesion Identification

To assess localization performance independently of the weakly supervised training signal as illustrated Section 5.2, we evaluated the model on a held-out subset of 100 test images annotated by four radiologists at Tikur Anbessa Specialized Hospital as illustrated in Figure 3 and Figure 6 on different cancer types and image modalities. These annotations were used exclusively for evaluation and were not accessible to the model at any stage of training or validation. On this expert-annotated subset, our model achieved a mean Intersection over Union (IoU) of 0.890, indicating Table 7 strong agreement between the model’s predicted disease-relevant regions and expert-identified ground truth, and supporting the clinical relevance of the model’s weakly supervised localization capability.

## 7. Conclusions

This study presented a weakly supervised multi-caption grounding framework for disease localization in medical images. The framework integrates multiple clinical captions using BioClinicalBERT and incorporates caption-importance weighting within a multimodal localization architecture. The approach was designed to use image-level supervision while exploiting information contained in multiple clinical descriptions. The model learns to prioritize primary diagnostic assertions while also taking into account clinical context. Experiments show significant agreement with expert-defined caption significance (0.9552 correlation) and better attention behavior over baselines (0.5011 vs. 0.4123, *p* < 0.01), while retaining competitive lesion identification performance (0.9811) with just image-level supervision. The model performs consistently across evaluation measures (0.9811 ± 0.010), demonstrating robustness and stability. These findings demonstrate the usefulness of fusing clinical report structure into vision-language models, which allows for better interpretable and clinically aligned disease localization in medical imaging.

The code used in this study is publicly available on our GitHub repository: LViT-BioClinicalBERT-Mutli-caption-Guided-Medical-image-localization (https://github.com/dawitemu1/LViT-BioClinicalBERT-Mutli-caption-Guided-Medical-image-localization-MCDL-, accessed on 29 August 2026).

## 8. Limitations and Future Directions

Our study LViT-BioClinicalBERT handles heterogeneous imaging data using a unified architecture that utilizes a modality-agnostic LViT encoder with caption-based textual conditioning via BioClinicalBER. This study reports aggregate performance across the combined dataset rather than by modality or cancer type. Future research will involve stratified, subgroup-level evaluations using statistical significance testing to further understand performance across modalities and cancer types.

Table 7: Although four radiologists were involved in the evaluation annotation process, we did not compute a formal inter-rater agreement statistic (e.g., mean pairwise IoU or Dice between annotators) across all four. As a result, we cannot fully quantify annotation consistency, and some subjectivity in lesion boundary delineation may remain. Future work should report a formal inter-rater agreement measure across all annotators to further strengthen confidence in the evaluation ground truth.

## Figures and Tables

**Figure 1:**
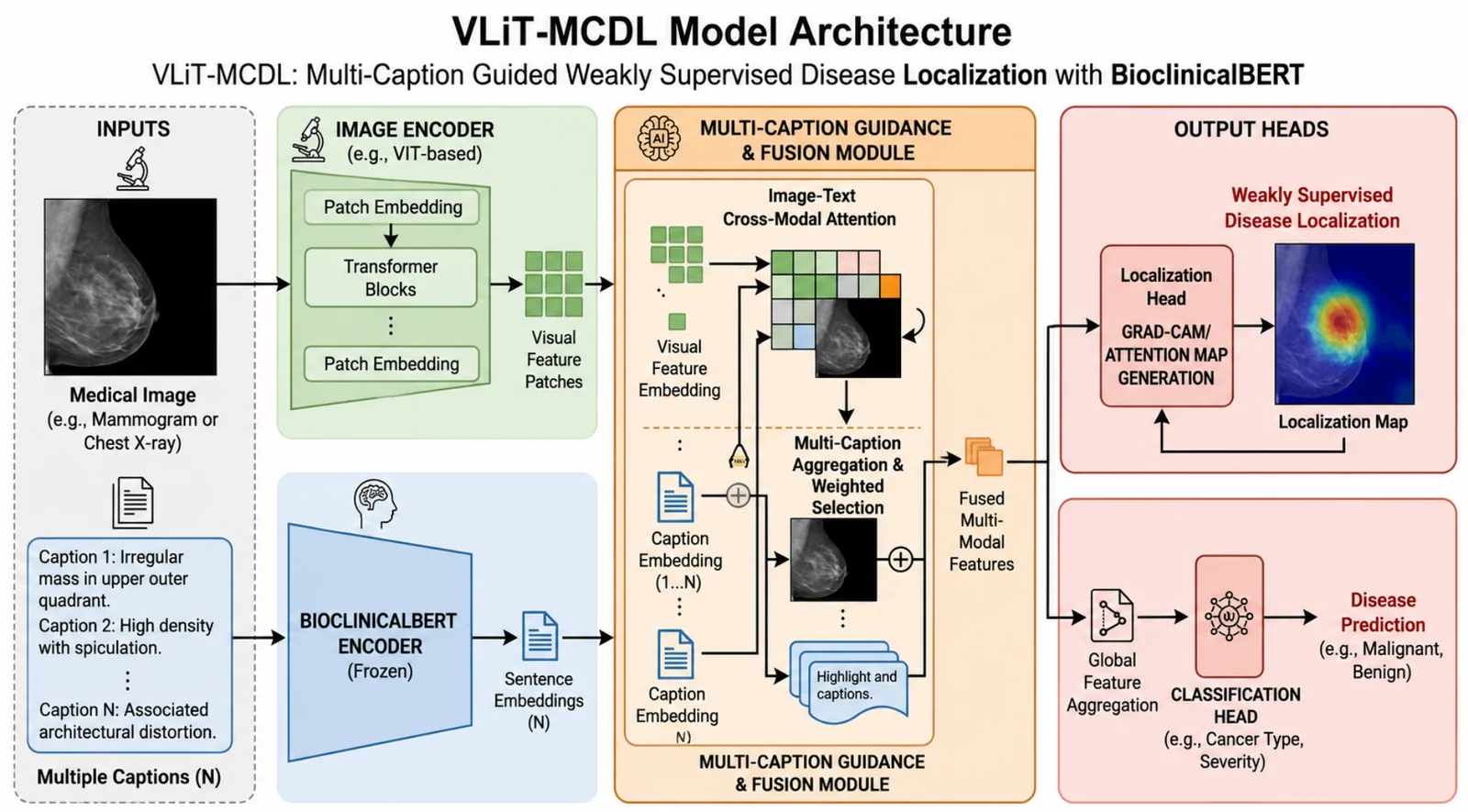
Proposed LViT-MCDL Framework: Multi-Caption Guided Weakly Supervised Disease Localization LViT with BioClinicalBERT.

**Figure 2:**
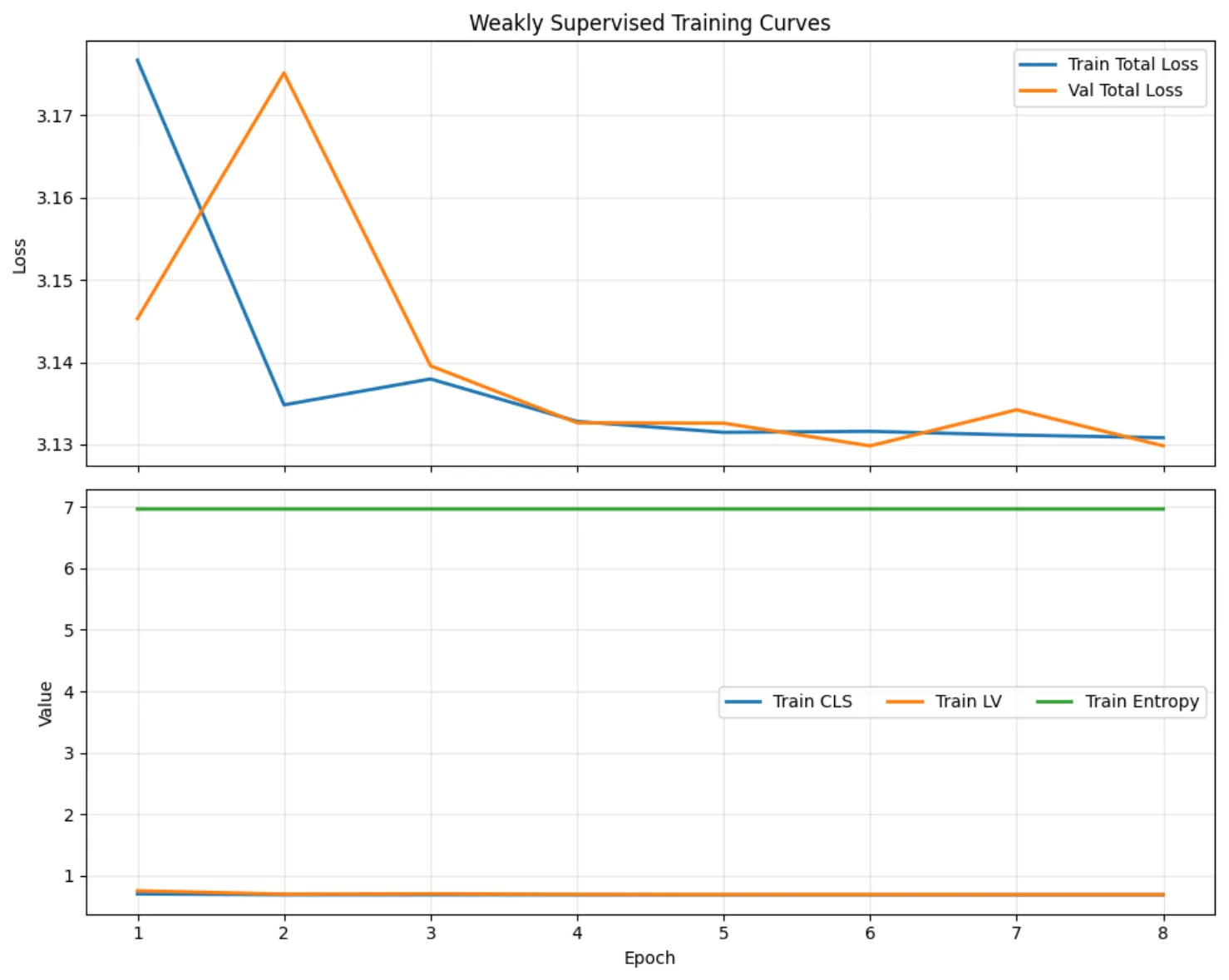
Weakly supervised training curves for LViT-BioClinicalBERT over 8 epochs. (**Top**) Training and validation total loss showing smooth convergence without overfitting. (**Bottom**) Component losses: classification (CLS), language-vision contrastive (LV), and entropy regularization. The model stabilizes after 4–5 epochs with consistent validation performance.

**Figure 3:**
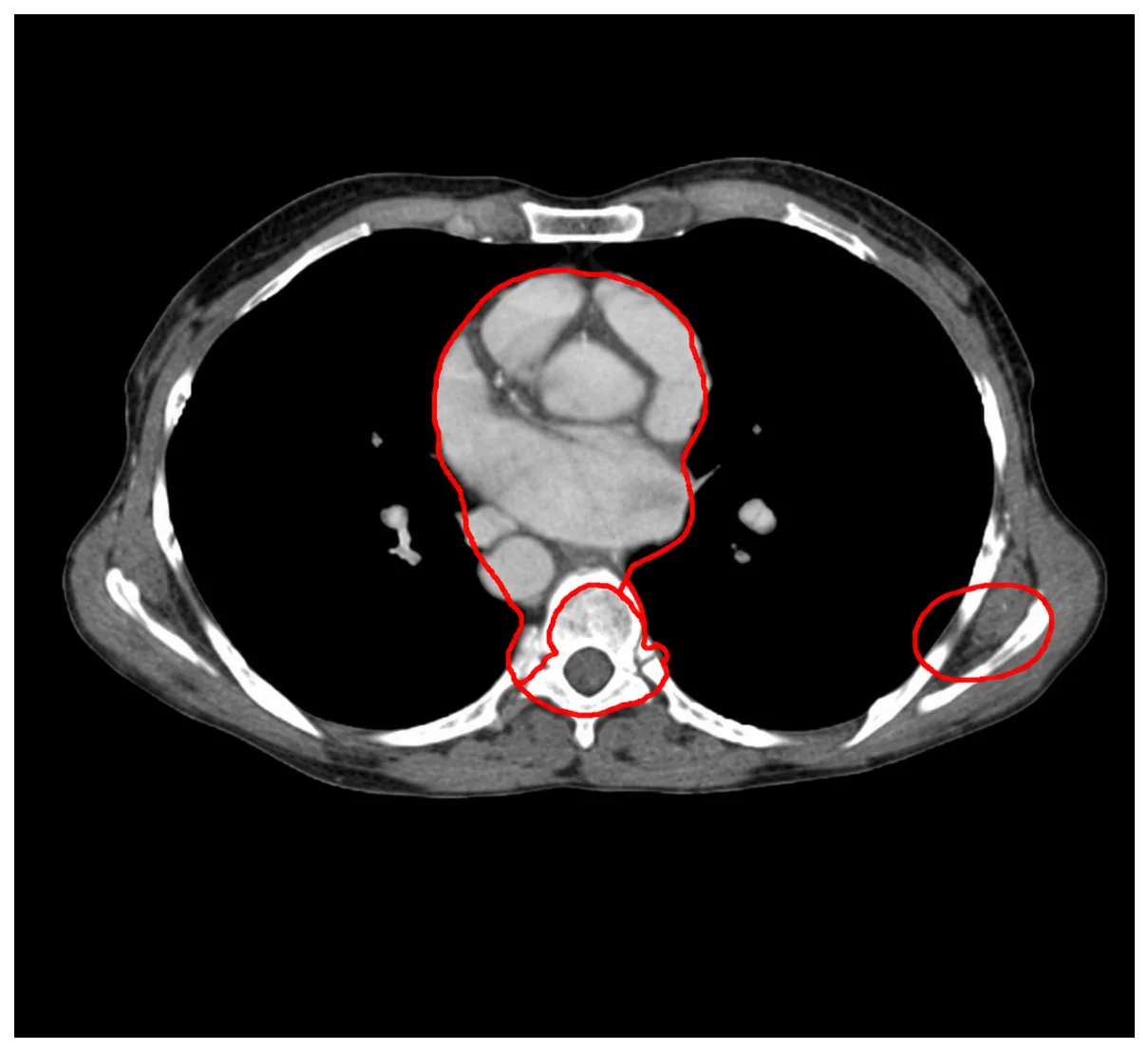
Representative cervical cancer MRI image from the 100-image expert-annotated test subset.

**Figure 4:**
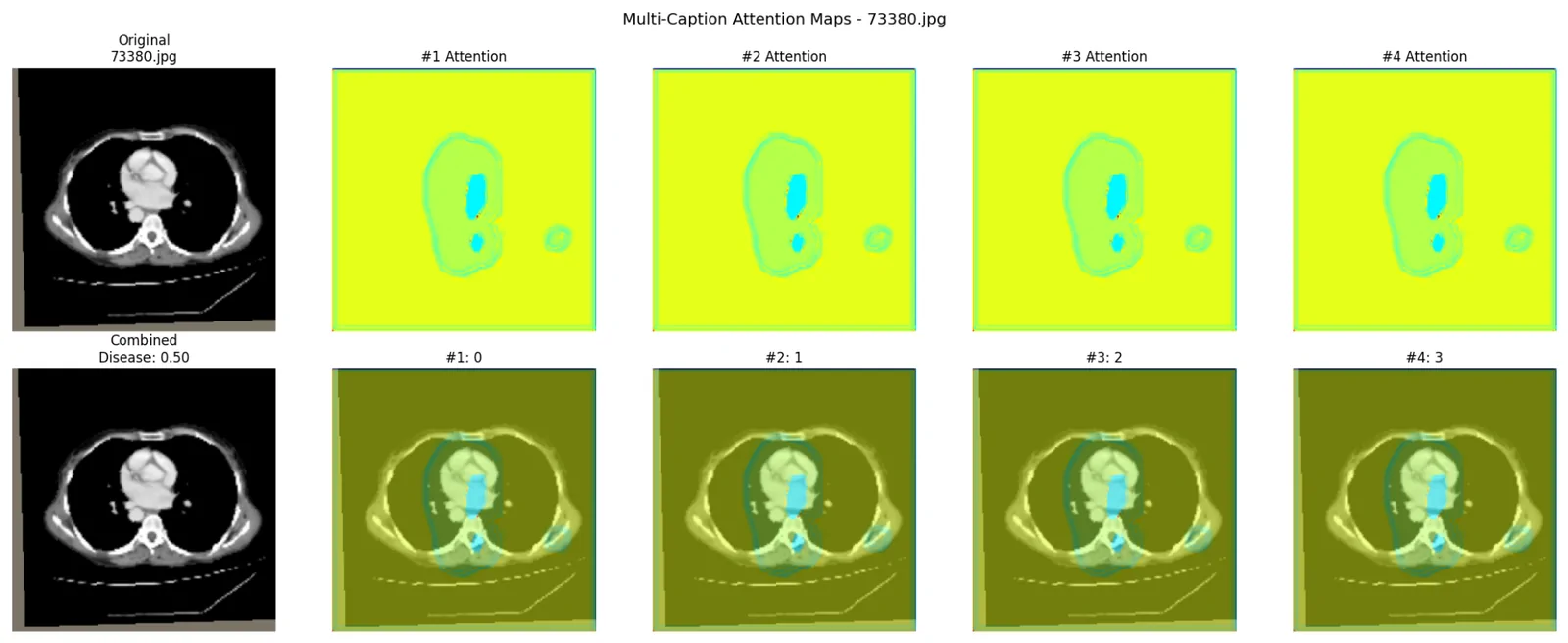
Disease region localization for the cervical cancer MRI case. From left to right: original image, attention heatmap, binary localization mask, region contours, attention distribution, and caption importance hierarchy.

**Figure 6:**
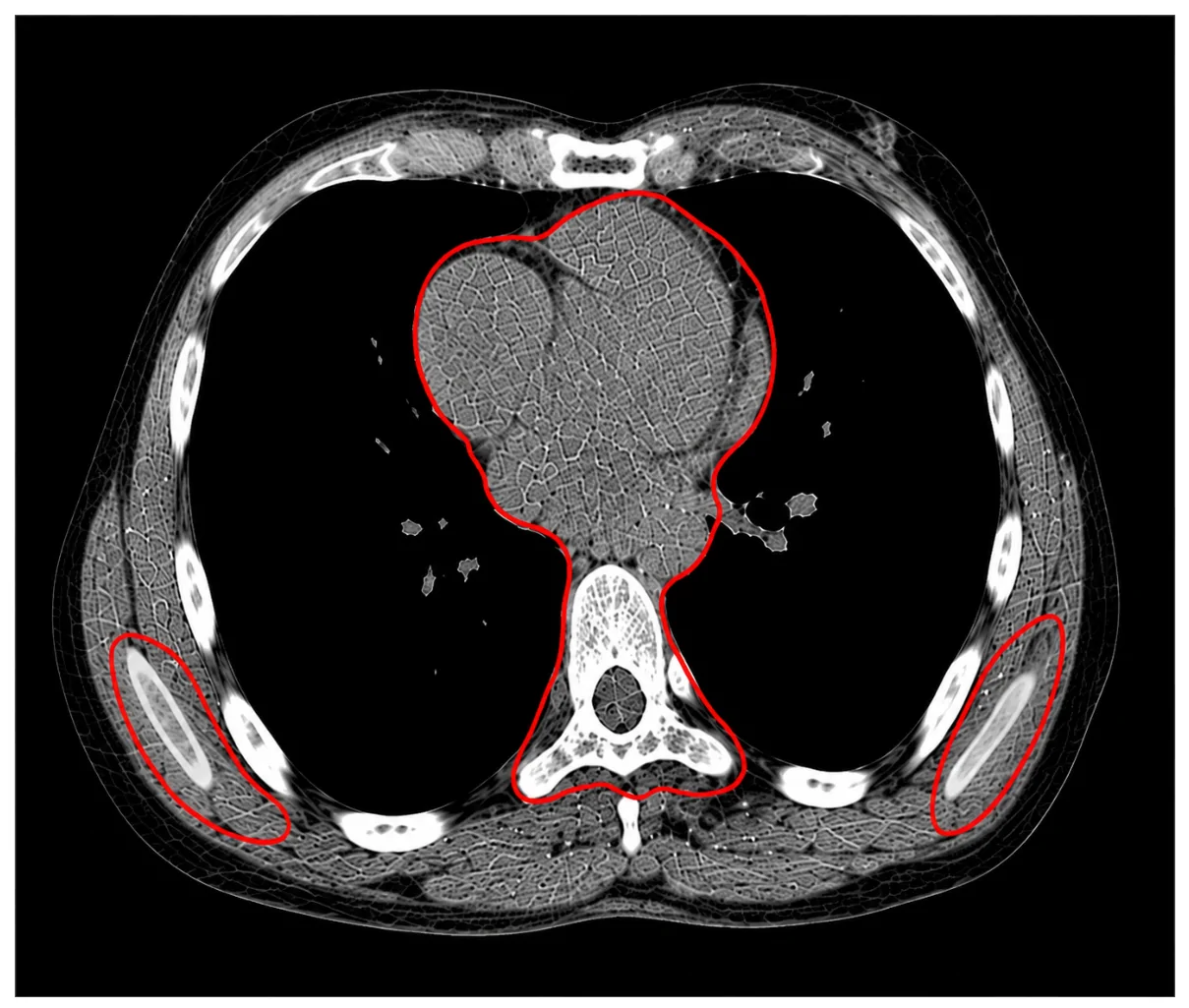
Representative liver cancer CT image from the 100-image expert-annotated test subset.

**Figure 7:**
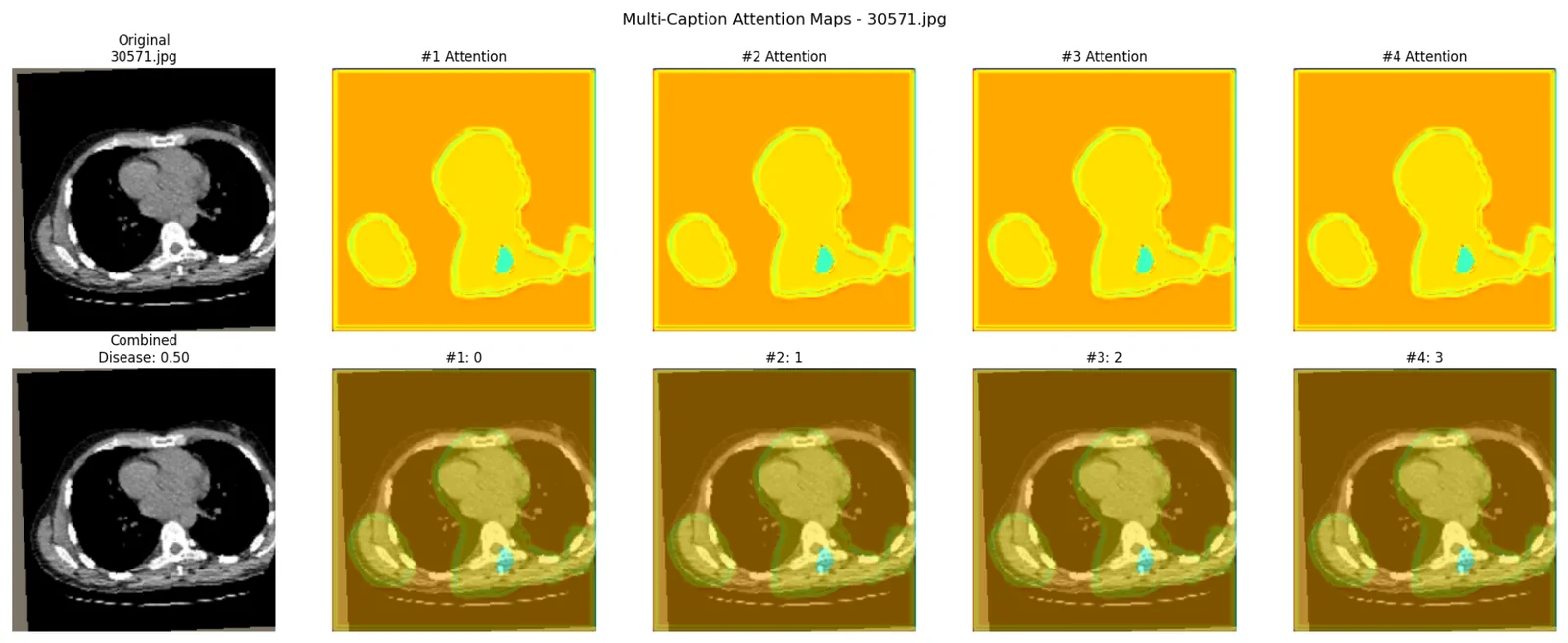
Disease region localization for the liver cancer CT case. From left to right: original image, attention heatmap, binary localization mask, region contours, attention distribution, and caption-importance hierarchy.

**Figure 8:**
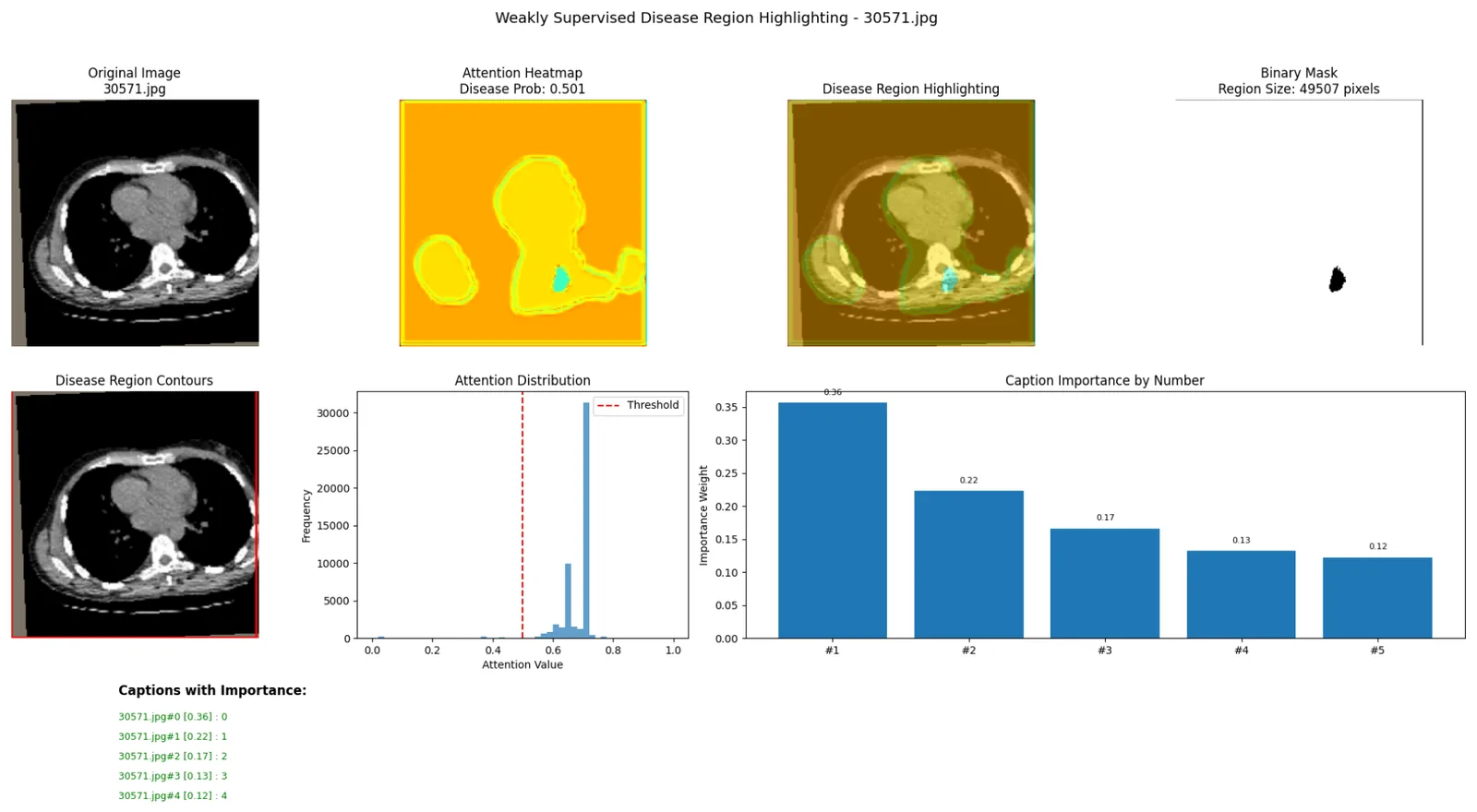
Multi-caption attention maps for the liver cancer CT case. Each subfigure shows the attention heatmap generated from an individual clinical caption in Figure A1. Each subfigure displays the attention heatmap corresponding to a specific clinical caption, as illustrated in Table 6.

**Table 1:** Example of caption–image mapping.

Image ID	Caption ID	Caption
100522.jpg	100522.jpg#0	startseq There is a right large heterogeneously enhancing upper lobe mass…
100522.jpg	100522.jpg#1	startseq There is a right superior sulcus mass leading to bony destruction…
100522.jpg	100522.jpg#2	startseq There is middle lobe bronchiectasis and nodularity…
100522.jpg	100522.jpg#3	startseq Conclusion Spinal canal narrowing as a result of bony invasion…
100522.jpg	100522.jpg#4	startseq Conclusion Known cervical cancer with multiple metastases…

**Table 2:** Mean and standard deviation of the training losses over five independent runs.

Metric	Mean ± Std
Training Loss	13.04±0.12
Validation Loss	13.01±0.15
Classification Loss	0.71±0.02
Localization Loss	0.68±0.03
Entropy Loss	7.00±0.00

**Table 3:** Comparison of weakly supervised medical imaging methods.

Models	Acc. (%)	CIC	AFS	AC	MDP
CNN and ResNet [12].	85–90	0.75	0.35	0.90	0.45
ViT-B/16/32, ViT-L/16/32, CaiT-xs24 [1].	90–95	0.85	0.45	0.92	0.48
CAN and ADN [5].	92–96	0.82	0.40	0.88	0.52
GLAS using ResNet50 [13].	85–97	—	—	—	—
CNN with a ViT [11].	95	0.80	0.42	0.87	**0.55**
DCNN [14].	88–92	0.70	0.30	0.85	0.42
ResNeet and ResNet [15].	90–93	0.73	0.33	0.86	0.44
GradCAM and GradCAM++ [30].	93–95	0.83	0.43	0.91	0.49
PLAM and LViT [18].	96	0.88	0.48	0.93	0.50
MedLangViT [23].	94–97	0.84	0.44	0.90	0.47
LViT and LV [18,30].	89–92	0.71	0.31	0.84	0.43
Our Model (LViT-BioClinicalBERT)	**98.11**	**0.9552**	**0.5006**	**0.9999**	0.5000

Note: Baseline values were obtained from the corresponding published studies and are provided for contextual comparison only. They were not reimplemented or evaluated under the experimental conditions of the present study.

**Table 4:** Component ablation study.

Variant	Importance Correlation	Attention Focus Score	lesion Accuracy
Full model	0.9552±0.0011	0.5006±0.0007	0.9811±0.1705
Without importance module	0.5000±0.0000 ***	0.5019±0.0008	0.6323±0.1409
Without consistency loss	0.9507±0.0011	0.5199±0.0125	0.5452±0.1703
Without sparsity loss	0.9507±0.0011	0.5011±0.0003	0.5447±0.1706
Single caption only	0.5000±0.0000 ***	0.5017±0.0007	0.6264±0.1722
Random caption order	0.9507±0.0011	0.5016±0.0006	0.9811±0.1704

Note: Statistical significance is indicated according to Section Abbreviations and Table A8. ***: Highly significant (p<0.001).

**Table 5:** Comparison of alignment strategies.

Alignment Strategy	Importance Correlation	Attention Focus Score	Lesion Accuracy
Full model (Our Alignment)	0.9552±0.0011	0.5006±0.0007	0.9811±0.1703
No alignment	0.5000±0.0000 ***	0.5019±0.0008	0.6326±0.1423
Cosine similarity	0.5798±0.0492 ***	0.4972±0.0032	0.4186±0.1602
Contrastive loss	0.5641±0.0493 ***	0.4975±0.0034	0.4228±0.1588
Alignment weight (α=0.1)	0.9163±0.0188	0.4972±0.0032	0.4219±0.1603
Alignment weight (α=0.3)	0.9527±0.0018	0.4971±0.0032	0.4259±0.1594
Alignment weight (α=0.5)	0.9507±0.0011	0.5017±0.0007	0.5459±0.1703
Alignment weight (α=0.7)	0.9519±0.0010	0.4969±0.0032	0.4266±0.1562
Alignment weight (α=1.0)	0.9507±0.0011	0.4968±0.0033	0.4072±0.1468

***: Highly significant (p<0.001).

**Table 7:** Four-expert evaluation with aggregated and overall results.

Metric	Expert 1	Expert 2	Expert 3	Expert 4	Overall (Mean ± Std)
Caption Importance Correlation	0.94	0.96	0.95	0.97	0.955±0.013
Attention Focus Score	0.49	0.51	0.50	0.50	0.500±0.008
Localization IoU	0.88	0.90	0.89	0.89	0.890±0.008
Classification Accuracy	0.9731	0.9731	0.9869	0.9869	0.980±0.008
Composite Score	0.72	0.74	0.73	0.74	0.733±0.010

**Figure A1:**
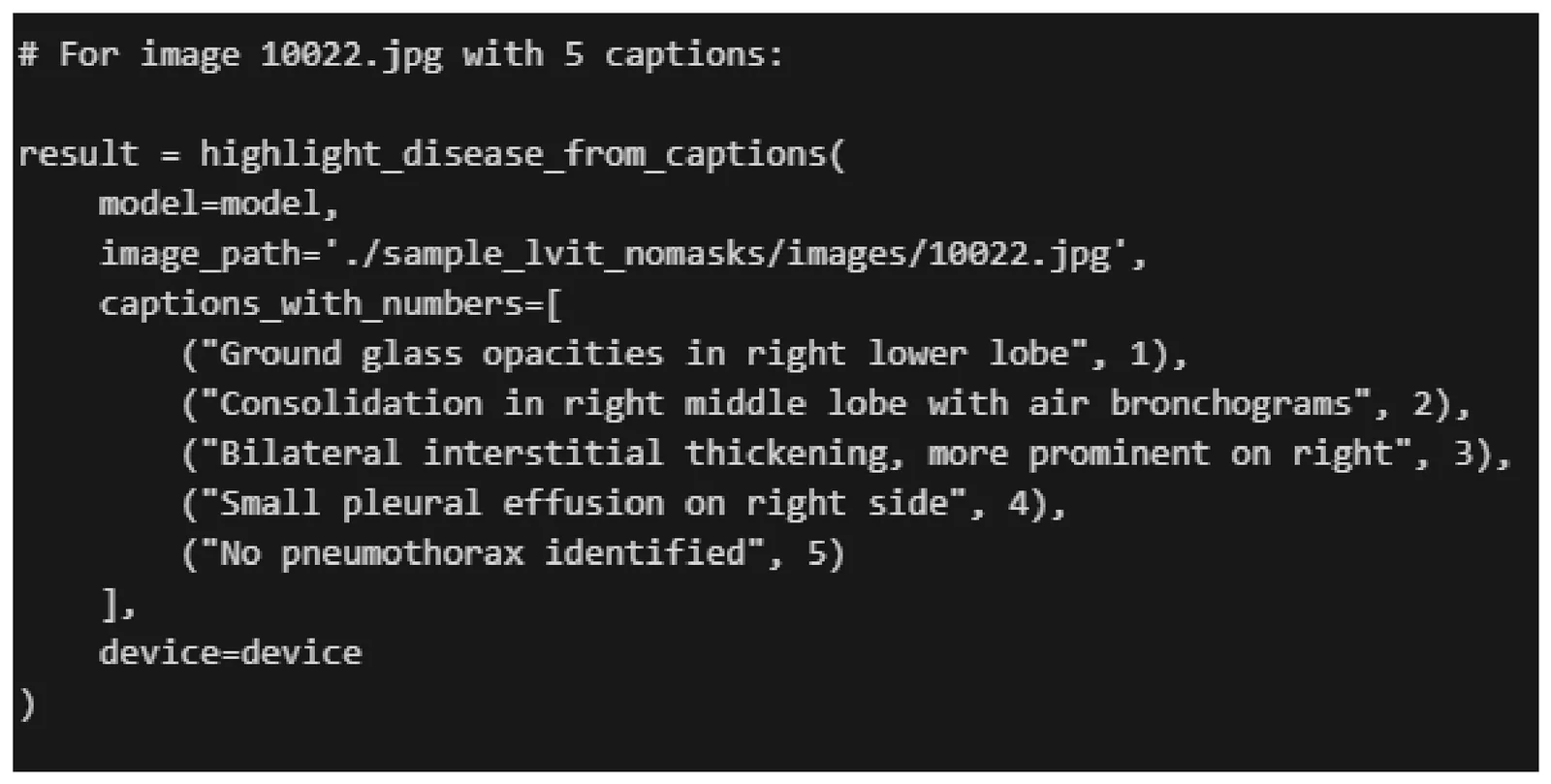
Example of multi-caption importance prediction for Image 10022.jpg with five clinical captions. The model assigns decreasing importance weights to captions based on clinical relevance: Caption #1 describing ground-glass opacities receives the highest weight, while supporting findings receive progressively lower weights. This demonstrates the model’s ability to learn clinical hierarchies without explicit supervision.

## Data Availability

Due to patient privacy and the rules of the centers’ institutional review boards, the datasets created and analyzed during this investigation are not publicly available; however, they can be obtained from the corresponding author upon reasonable request.

## Abbreviations

The following abbreviations are used in this manuscript:

***	Highly significant (p<0.001)
**	Very significant (p<0.01)
*	Significant (p<0.05)
ns	Not significant (p≥0.05)
w/	With
w/o	Without
LViT	Language meets Vision Transformer
MRN	Medical Record Number
ViT	Vision Transformer
CLS	Classification loss
LV	Language-Vision contrastive loss
IoU	Intersection over Union
CAM	lass Activation Mapping
Acc.	Classification Accuracy
CIC	Caption Importance Correlation
AFS	Attention Focus Score
AC	Attention Consistency
MDP	Mean Disease Probability
CAN	Confidence Aware Network
ADN	Anomaly Detection Network
PLAM	Pixel Level Attention Module

## Appendix A. Component Ablation Details

Table A8: This table presents the criticality ranking of each component in our model based on statistical significance. ******* indicates *p* < 0.001 (highly significant), ns indicates not significant (*p* ≥ 0.05). The importance module is the only component marked as **essential** because removing it causes a highly significant drop in importance correlation (*p* < 0.001). All other components (*w*/*o* consistency loss, *w*/*o sparsity loss*, *single caption only*, *random caption order*) show no statistically significant impact on any metric, indicating the model remains **robust** to their removal. N/A indicates that importance correlation cannot be computed for single-caption models, as there is only one caption to weight.

**Table A1:** Detailed Statistical Analysis: Component Ablation—Importance Correlation.

Variant	Score ± SE	Δ from Full	t-Statistic	*p*-Value	Significance
Full model	0.9507±0.0011	—	—	—	—
w/o importance module	0.5000±0.0000	−0.4507	−409.48	$1.37\times 10^{-16}$	***
w/o consistency loss	0.9507±0.0011	0.0000	0.00	1.00	ns
w/o sparsity loss	0.9507±0.0011	0.0000	0.00	1.00	ns
Single caption only	0.5000±0.0000	−0.4507	−409.48	$1.37\times 10^{-16}$	***
Random caption order	0.9507±0.0011	0.0000	0.00	1.00	ns

***: Highly significant (p<0.001). ns: Not significant (p≥0.05).

**Table A2:** Detailed Statistical Analysis: Component Ablation—Attention Focus Score.

Variant	Score ± SE	Δ from Full	t-Statistic	*p*-Value	Significance
Full model	0.5017±0.0007	—	—	—	—
w/o importance module	0.5019±0.0008	0.0002	0.22	$8.32\times 10^{-1}$	ns
w/o consistency loss	0.5199±0.0125	0.0182	1.45	$1.89\times 10^{-1}$	ns
w/o sparsity loss	0.5011±0.0003	−0.0005	−0.75	$4.71\times 10^{-1}$	ns
Single caption only	0.5017±0.0007	0.0000	0.03	$9.74\times 10^{-1}$	ns
Random caption order	0.5016±0.0006	−0.0000	−0.02	$9.86\times 10^{-1}$	ns

ns: Not significant (p≥0.05).

**Table A3:** Detailed Statistical Analysis: Component Ablation—Classification Accuracy.

Variant	Score ± SE	Δ from Full	t-Statistic	*p*-Value	Significance
Full model	0.5457±0.1705	—	—	—	—
w/o importance module	0.6323±0.1409	0.0855	0.39	$7.05\times 10^{-1}$	ns
w/o consistency loss	0.5452±0.1703	−0.0017	−0.01	$9.95\times 10^{-1}$	ns
w/o sparsity loss	0.5447±0.1706	−0.0021	−0.01	$9.93\times 10^{-1}$	ns
Single caption only	0.6264±0.1722	0.0795	0.33	$7.47\times 10^{-1}$	ns
Random caption order	0.5457±0.1704	−0.0012	−0.00	$9.96\times 10^{-1}$	ns

ns: Not significant (p≥0.05).

## Appendix B. Alignment Strategies Details

Table A7: This table ranks alignment strategies by their composite score, which is the average of the three normalized metrics (Importance Correlation, Attention Focus Score, and Classification Accuracy) and evaluation details Table A4, Table A5 and Table A6. Our proposed alignment with α=0.5 achieves the highest composite score (0.6661), demonstrating the optimal balance across all metrics. While α=0.3 and α=0.7 show slightly higher importance correlation (0.9527 and 0.9519 respectively), they sacrifice focus score and classification accuracy. No alignment achieves the highest classification accuracy (0.6326) but fails completely on importance correlation (0.5000, random guessing), confirming that alignment is essential for learning clinical hierarchies. Simple approaches like cosine similarity and contrastive loss perform poorly across all metrics, validating the effectiveness of our learned alignment mechanism.

**Table A4:** Detailed Statistical Analysis: Alignment Strategies—Importance Correlation.

Alignment Strategy	Score ± SE	Δ from Full	t-Statistic	*p*-Value	Significance
Full model (Our Alignment)	0.9507±0.0011	—	—	—	—
No alignment	0.5000±0.0000	−0.4507	−409.48	$1.37\times 10^{-16}$	***
Cosine similarity	0.5798±0.0492	−0.3709	−7.53	$1.33\times 10^{-4}$	***
Contrastive loss	0.5641±0.0493	−0.3866	−7.85	$1.02\times 10^{-4}$	***
Alignment weight (α=0.1)	0.9163±0.0188	−0.0344	−1.83	$1.10\times 10^{-1}$	ns
Alignment weight (α=0.3)	0.9527±0.0018	0.0020	0.92	$3.74\times 10^{-1}$	ns
Alignment weight (α=0.7)	0.9519±0.0010	0.0013	0.84	$4.14\times 10^{-1}$	ns
Alignment weight (α=1.0)	0.9507±0.0011	0.0000	0.00	1.00	ns

***: Highly significant (p<0.001). ns: Not significant (p≥0.05).

**Table A5:** Detailed Statistical Analysis: Alignment Strategies—Attention Focus Score.

Alignment Strategy	Score ± SE	Δ from Full	t-Statistic	*p*-Value	Significance
Full model (Our Alignment)	0.5017±0.0007	—	—	—	—
No alignment	0.5019±0.0008	0.0002	0.21	$8.38\times 10^{-1}$	ns
Cosine similarity	0.4972±0.0032	−0.0045	−1.37	$2.11\times 10^{-1}$	ns
Contrastive loss	0.4975±0.0034	−0.0042	−1.20	$2.67\times 10^{-1}$	ns
Alignment weight (α=0.1)	0.4972±0.0032	−0.0045	−1.37	$2.10\times 10^{-1}$	ns
Alignment weight (α=0.3)	0.4971±0.0032	−0.0046	−1.39	$2.03\times 10^{-1}$	ns
Alignment weight (α=0.7)	0.4969±0.0032	−0.0048	−1.44	$1.89\times 10^{-1}$	ns
Alignment weight (α=1.0)	0.4968±0.0033	−0.0049	−1.48	$1.80\times 10^{-1}$	ns

ns: Not significant (p≥0.05).

**Table A6:** Detailed Statistical Analysis: Alignment Strategies—Classification Accuracy.

Alignment Strategy	Score ± SE	Δ from Full	t-Statistic	*p*-Value	Significance
Full model (Our Alignment)	0.5459±0.1703	—	—	—	—
No alignment	0.6326±0.1423	0.0857	0.39	$7.05\times 10^{-1}$	ns
Cosine similarity	0.4186±0.1602	−0.1283	−0.55	$5.91\times 10^{-1}$	ns
Contrastive loss	0.4228±0.1588	−0.1241	−0.53	$6.02\times 10^{-1}$	ns
Alignment weight (α=0.1)	0.4219±0.1603	−0.1250	−0.54	$6.01\times 10^{-1}$	ns
Alignment weight (α=0.3)	0.4259±0.1594	−0.1210	−0.52	$6.12\times 10^{-1}$	ns
Alignment weight (α=0.7)	0.4266±0.1562	−0.1203	−0.52	$6.10\times 10^{-1}$	ns
Alignment weight (α=1.0)	0.4072±0.1468	−0.1397	−0.62	$5.44\times 10^{-1}$	ns

ns: Not significant (p≥0.05).

**Table A7:** Alignment Strategy: Performance Ranking.

Alignment Strategy	Importance Correlation	Focus Score	Class. Accuracy	Composite
Full model (Our Alignment)	0.9507	0.5017	0.5459	0.6661
Alignment weight (α=0.3)	0.9527	0.4971	0.4259	0.6252
Alignment weight (α=0.7)	0.9519	0.4969	0.4266	0.6252
Alignment weight (α=1.0)	0.9507	0.4968	0.4072	0.6182
Alignment weight (α=0.1)	0.9163	0.4972	0.4219	0.6118
No alignment	0.5000	0.5019	0.6326	0.5448
Cosine similarity	0.5798	0.4972	0.4186	0.4985
Contrastive loss	0.5641	0.4975	0.4228	0.4948

**Table A8:** Summary Heatmap: Statistical Significance.

Variant	Importance Correlation	Attention Focus	Classification
Full model	0.9507	0.5017	0.5457
w/o importance module	0.5000	0.5019	0.6323
w/o consistency loss	0.9507	0.5199	0.5452
w/o sparsity loss	0.9507	0.5011	0.5447
Single caption only	0.5000	0.5017	0.6264
Random caption order	0.9507	0.5016	0.5457
Full model (Our Alignment)	0.9507	0.5017	0.5459
No alignment	0.5000	0.5019	0.6326
Cosine similarity	0.5798	0.4972	0.4186
Contrastive loss	0.5641	0.4975	0.4228
Alignment weight (α=0.1)	0.9163	0.4972	0.4219
Alignment weight (α=0.3)	0.9527	0.4971	0.4259
Alignment weight (α=0.7)	0.9519	0.4969	0.4266
Alignment weight (α=1.0)	0.9507	0.4968	0.4072

## References

[1] Luo X., Xu S., Xie Y., Hu J., He Y., Deng Y., Huang H. (2025). TWLR: Text-Guided Weakly-Supervised Lesion Localization and Severity Regression for Explainable Diabetic Retinopathy Grading. arXiv.

[2] Keppeler V., Lederer M., Leucht U.A. (2022). Explainable Artificial Intelligence. Encyclopedia of Data Science and Machine Learning.

[3] Guo Y., He Y., Lyu J., Zhou Z., Yang D., Ma L., Tan H., Chen C., Zhang W., Hu J. (2022). Deep Learning with Weak Annotation from Diagnosis Reports for Detection of Multiple Head Disorders: A Prospective, Multicentre Study. Lancet Digit. Health.

[4] Shoaib M.R., Niyato D., Emara H.M., Zhao J. (2025). Automated Segmentation and Classification of Brain Magnetic Resonance Images for Brain Tumor Diagnosis Based on Deep Learning. IEEE Internet Things J..

[5] Kabiraj A., Meena T., Tadepalli K., Roy S. (2024). An Explainable Weakly Supervised Model for Multi-Disease Detection and Localization from Thoracic X-Rays. Appl. Soft Comput..

[6] Roy S., Bhattacharyya D., Bandyopadhyay S.K., Kim T.H. (2017). An Effective Method for Computerized Prediction and Segmentation of Multiple Sclerosis Lesions in Brain MRI. Comput. Methods Programs Biomed..

[7] Koutoulakis E., Trivizakis E., Markodimitrakis E., Agelaki S., Tsiknakis M., Marias K. (2026). A Critical Review of Explainable Deep Learning in Lung Cancer Diagnosis. Artif. Intell. Rev..

[8] Simonyan K., Zisserman A. Very Deep Convolutional Networks for Large-Scale Image Recognition. Proceedings of the 3rd International Conference on Learning Representations (ICLR).

[9] Li D., Huo H., Jiao S., Sun X., Chen S. (2024). Automated Thorax Disease Diagnosis Using Multi-Branch Residual Attention Network. Sci. Rep..

[10] Wang T., Huang K., Xu M., Huang J. (2024). Weakly Supervised Chest X-Ray Abnormality Localization with Non-Linear Modulation and Foreground Control. Sci. Rep..

[11] Li F., Zhou L., Wang Y., Chen C., Yang S., Shan F., Liu L. (2022). Modeling Long-Range Dependencies for Weakly Supervised Disease Classification and Localization on Chest X-Ray. Quant. Imaging Med. Surg..

[12] Hermoza R., Maicas G., Nascimento J.C., Carneiro G. (2020). Region Proposals for Saliency Map Refinement for Weakly-Supervised Disease Localisation and Classification. Medical Image Computing and Computer Assisted Intervention—MICCAI 2020.

[13] Rony J., Belharbi S., Dolz J., Ben Ayed I., McCaffrey L., Granger E. (2023). Deep Weakly-Supervised Learning Methods for Classification and Localization in Histology Images: A Survey. Mach. Learn. Biomed. Imaging.

[14] Wang X., Peng Y., Lu L., Lu Z., Bagheri M., Summers R.M. (2019). ChestX-ray: Hospital-Scale Chest X-ray Database and Benchmarks on Weakly Supervised Classification and Localization of Common Thorax Diseases. Advances in Computer Vision and Pattern Recognition.

[15] Yao L., Prosky J., Poblenz E., Covington B., Lyman K. (2018). Weakly Supervised Medical Diagnosis and Localization from Multiple Resolutions. arXiv.

[16] Grassucci E., Cicchetti G., Comminiello D. Closing the Gap in Multimodal Medical Representation Alignment. Proceedings of the IEEE International Workshop on Machine Learning for Signal Processing (MLSP).

[17] Izhar A., Japar N., Idris N., Dang T. MicarVLMoE: A Modern Gated Cross-Aligned Vision-Language Mixture of Experts Model for Medical Image Captioning and Report Generation. Proceedings of the International Joint Conference on Neural Networks (IJCNN).

[18] Li Z., Li Y., Li Q., Wang P., Guo D., Lu L., Jin D., Zhang Y., Hong Q. (2024). LViT: Language Meets Vision Transformer in Medical Image Segmentation. IEEE Trans. Med. Imaging.

[19] Bekker J., Davis J. (2020). Learning from Positive and Unlabeled Data: A Survey. Mach. Learn..

[20] Yu K., Ghosh S., Liu Z., Deible C., Batmanghelich K. (2022). Anatomy-Guided Weakly-Supervised Abnormality Localization in Chest X-rays. Medical Image Computing and Computer Assisted Intervention—MICCAI 2022.

[21] Bhalodia R., Hatamizadeh A., Tam L., Xu Z., Wang X., Turkbey E., Xu D. (2021). Improving Pneumonia Localization via Cross-Attention on Medical Images and Reports. Medical Image Computing and Computer Assisted Intervention—MICCAI 2021.

[22] Wadden D., Wennberg U., Luan Y., Hajishirzi H. Entity, Relation, and Event Extraction with Contextualized Span Representations. Proceedings of the 2019 Conference on Empirical Methods in Natural Language Processing and 9th International Joint Conference on Natural Language Processing (EMNLP-IJCNLP).

[23] Wang Y., Su J., Li X. (2025). MedLangViT: A Language–Vision Network for Medical Image Segmentation. Electronics.

[24] Wang J., Xu J., Zhou Y., Luo X., Wang H., Wang T., Li H. (2026). MultiXpert: Dual-Stream Synergistic Enhancement with Cross-Modal Alignment for Zero-Shot Chest X-Ray Diagnosis. Inf. Process. Manag..

[25] Li S.-Q., Zhen C., Li B.-Q., Zhao J.-G., Huang J.-S. (2026). Sparsity-Guided Multimodal Self-Supervised Learning for Expert-Level Multi-Task Chest X-Ray Diagnosis. Expert Syst. Appl..

[26] Yang D., Zou Y.X., Zhang J., Li G. (2021). GID-Net: Detecting Human-Object Interaction with Global and Instance Dependency. Neurocomputing.

[27] Almughamisi N., Abosamra G., Albar A., Saleh M. (2026). Anatomy-Guided Hybrid CNN–ViT Model with Neuro-Symbolic Reasoning for Early Diagnosis of Thoracic Diseases Multilabel. Diagnostics.

[28] Pan Q., Li Z., Yang G., Yang Q., Ji B. (2025). EviVLM: When Evidential Learning Meets Vision Language Model for Medical Image Segmentation. IEEE Trans. Med. Imaging.

[29] Zhi T., Liu H., Wang X., Ibrahim U.M., Meng C. (2026). MultimodalCNN-PD: A Parkinson’s Disease Diagnostics Framework Using Multimodal Convolutional Neural Network. Front. Aging Neurosci..

[30] Yan C., Yao J., Li R., Xu Z., Huang J. Weakly Supervised Deep Learning for Thoracic Disease Classification and Localization on Chest X-rays. Proceedings of the 2018 ACM International Conference on Bioinformatics, Computational Biology, and Health Informatics (ACM-BCB ’18).

[31] Ge S., Chen Q., Jiang Z., Yin Y., Qin L., Chen Z., Gu Q. (2025). Implicit Location-Caption Alignment via Complementary Masking for Weakly-Supervised Dense Video Captioning. Proc. AAAI Conf. Artif. Intell..

[32] Yang H., Zhou H.Y., Liu J., Huang W., Li C., Li Z., Gao Y., Liu Q., Liang Y., Yang Q. (2026). A multimodal vision–language model for generalizable annotation-free pathology localization. Nat. Biomed. Eng..

[33] Lu Y., Wang A. (2025). Integrating language into medical visual recognition and reasoning: A survey. Med. Image Anal..

[34] Li X., Li L., Li M., Yan P., Feng T., Luo H., Zhao Y., Yin S. (2026). Knowledge distillation and teacher–student learning in medical imaging: Comprehensive overview, pivotal role, and future directions. Med. Image Anal..

[35] Li X., Sun M., Wang Z., Han B., Wang Y., Wang Z., Zhao Y., Feng T. (2026). Optical fiber intelligent carpet for gait recognition with a local strain and global contour dual-modality attention network. IEEE Internet Things J..

[36] Sun Y., Li X., Li L., Feng T., Zhao Y., Yin S. (2026). PHH-FL: Perceptual hashing hypernetwork personalized federated learning for heterogeneous medical image analysis tasks. IEEE Internet Things J..

[37] Lozano A., Sun M.W., Burgess J., Chen L., Nirschl J.J., Gu J., Lopez I., Aklilu J., Rau A., Katzer A.W. BIOMEDICA: An Open Biomedical Image-Caption Archive, Dataset, and Vision-Language Models Derived from Scientific Literature. Proceedings of the IEEE/CVF Conference on Computer Vision and Pattern Recognition (CVPR).

[38] Zhang Y., Gao J., Zhou M., Wang X., Qiao Y., Zhang S., Wang D. (2023). Text-Guided Foundation Model Adaptation for Pathological Image Classification. Medical Image Computing and Computer Assisted Intervention—MICCAI 2023.

[39] Kuling G., Curpen B., Martel A.L. (2022). BI-RADS BERT and Using Section Segmentation to Understand Radiology Reports. J. Imaging.

[40] Kollapally N.M., Geller J. (2023). Clinical BioBERT Hyperparameter Optimization using Genetic Algorithm. arXiv.

[41] BioClinicalBERT: Domain-Specific Clinical NLP. https://www.emergentmind.com/topics/bioclinicalbert.

[42] Hossain M.R., Mahabub S., Al Masum A., Jahan I. (2024). Natural Language Processing (NLP) in Analyzing Electronic Health Records for Better Decision Making. J. Comput. Sci. Technol. Stud..

[43] Adahada E., Sassoon I., Hone K., Li Y. A Fully Transformer-Based Multimodal Framework for Explainable Breast Cancer Image Segmentation Using Radiology Reports. Proceedings of the International Conference on Computer Vision and Data Mining (ICCVDM).

[44] Dosovitskiy A., Beyer L., Kolesnikov A., Weissenborn D., Zhai X., Unterthiner T., Dehghani M., Minderer M., Heigold G., Gelly S. An Image Is Worth 16×16 Words: Transformers for Image Recognition at Scale. Proceedings of the International Conference on Learning Representations (ICLR).

[45] Lee J., Yoon W., Kim S., Kim D., Kim S., So C.H., Kang J. (2020). BioBERT: A Pre-Trained Biomedical Language Representation Model for Biomedical Text Mining. Bioinformatics.

[46] Alsentzer E., Murphy J.R., Boag W., Weng W.-H., Jin D., Naumann T., McDermott M.B.A. (2019). Publicly Available Clinical BERT Embeddings. arXiv.

[47] Zhang Y., Yang Q. (2017). A Survey on Multi-Task Learning. arXiv.

[48] Zhang C., Liu J., Dang K., Zhang W. Multi-Scale Distillation from Multiple Graph Neural Networks. Proceedings of the 36th AAAI Conference on Artificial Intelligence (AAAI).

[49] Zhou B., Khosla A., Lapedriza A., Oliva A., Torralba A. Learning Deep Features for Discriminative Localization. Proceedings of the IEEE Conference on Computer Vision and Pattern Recognition (CVPR).

[50] Zhang Q. (2022). A Novel ResNet101 Model Based on Dense Dilated Convolution for Image Classification. SN Appl. Sci..

[51] Wen C., Ye M., Li H., Chen T., Xiao X. (2025). Concept-Based Lesion Aware Transformer for Interpretable Retinal Disease Diagnosis. IEEE Trans. Med. Imaging.

[52] Yu S., Ma K., Bi Q., Bian C., Ning M., He N., Li Y., Liu H., Zheng Y. (2021). MIL-VT: Multiple Instance Learning Enhanced Vision Transformer for Fundus Image Classification. Medical Image Computing and Computer Assisted Intervention—MICCAI 2021.

[53] Huang Y., Lin L., Cheng P., Lyu J., Tang X. (2021). Lesion-Based Contrastive Learning for Diabetic Retinopathy Grading from Fundus Images. Medical Image Computing and Computer Assisted Intervention—MICCAI 2021.

[54] Sounack T., Davis J., Durieux B., Chaffin A., Pollard T.J., Lehman E., Johnson A.E.W., McDermott M., Naumann T., Lindvall C. (2025). BioClinical ModernBERT: A State-of-the-Art Long-Context Encoder for Biomedical and Clinical NLP. arXiv.

[55] Wang L., Yang N., Huang X., Jiao B., Yang L., Jiang D., Majumder R., Wei F. (2024). Text Embeddings by Weakly-Supervised Contrastive Pre-training. arXiv.

[56] M M.M., Mahesh T.R., V V.K., Guluwadi S. (2024). Enhancing Brain Tumor Detection in MRI Images through Explainable AI Using Grad-CAM with ResNet50. BMC Med. Imaging.

